# Open Access Scheduling for Patients at High Risk of Hospitalization and Incarceration

**DOI:** 10.7759/cureus.115253

**Published:** 2026-08-26

**Authors:** Abdullah J Allawala, Ana Turner

**Affiliations:** 1 Psychiatry and Behavioral Sciences, University of Florida College of Medicine, Jacksonville, Jacksonville, USA; 2 Psychiatry and Behavioral Sciences, University of Florida College of Medicine, Gainesville, USA

**Keywords:** assertive community treatment, community psychiatry, open access scheduling, severe mental illness, street psychiatry

## Abstract

Objective: To discuss the origins of assertive community treatment (ACT) and the adoption of its framework by a Northeast Florida Community Outreach Center, Sulzbacher, through the Mental Health Offender Program (MHOP). With recent changes to health policy and resource funding, MHOP implemented an alternative approach to providing patient care through Open Access Scheduling (OAS). This article examines the hypothesis that OAS may improve the outcomes on rates of missed long-acting injectable (LAI) antipsychotics, overdue psychiatric appointments, and incarceration.

Method: A retrospective comparative analysis was conducted among participants of the MHOP 12 months before and 12 months after implementation of OAS. With Institutional Review Board (IRB) exemption status granted, de-identified data obtained from the electronic medical record and publicly available arrest rates were compiled into a Microsoft Excel spreadsheet and subjected to statistical analysis using the Data ToolPak software. Primary outcomes measured were the percentage of overdue psychiatric appointments and missed LAI antipsychotics, and rates of incarceration before and after the implementation of OAS.

Results: Using a paired t-test design, the mean weekly percent of overdue psychiatric appointments and missed LAI antipsychotics increased from 51.7% pre-intervention to 57.0% post-intervention with a t-value of approximately 2.22 and a statistically significant p-value (0.032). However, rates on incarceration remained roughly the same. An unpaired t-test, assuming unequal variances, yielded a t-value of approximately 0.26 and a non-statistically significant p-value of 0.796.

Conclusion: Data analysis of 52 weeks before and after the implementation of OAS highlights the potential role of OAS as a viable alternative to psychiatric follow-up.

## Introduction

Background

Assertive community treatment (ACT) is a community-based service delivery model designed for individuals with severe and persistent mental illness who struggle with traditional outpatient care, frequently visit hospitals/emergency rooms, or are at risk of homelessness or incarceration [1]. ACT has been found to be more effective than standard services in reducing hospital use and increasing community tenure, and some studies have found improvements in stable housing, symptom management, and quality of life [2]. ACT consists of a multidisciplinary team of 10-12 mental health and rehabilitation professionals who work collaboratively to deliver comprehensive services in the places and contexts where they are needed (i.e., in homes, on the streets). Historically, ACT programs have been funded through federal grants and public insurance programs, including Medicaid, and typical models involved one full-time psychiatrist for every 100 patients [1].

In the 1990s, through philanthropic efforts in Northeast Florida, Sulzbacher was opened to serve the needs of the homeless population. In 2001, Sulzbacher was designated as a Federally Qualified Health Center and expanded its social outreach service. What began as an initiative to provide meals and basic medical services evolved over the decades as a shelter for men, women, and children, with broadened medical care including dental and behavioral health. Building on this evolution and in an effort to provide cost-effective care, Sulzbacher expanded its outreach services to assist those with mental illness at high risk and hospitalization and incarceration.

Implementation of an ACT-like jail diversion program

ACT funding and availability vary by state, often driven by Medicaid reimbursement rates, state general funds, and federal grants. Its availability remains limited in many areas, with only about 13% of mental health facilities providing it in 2015, and of those, less than 20% reported offering all its core services [3]. In 2021, thanks to the funding from multiple community partners, Sulzbacher implemented the Mental Health Offender Program (MHOP), resembling an ACT-like model with a "boots on the ground approach" of meeting patients where they are. Similar to ACT, MHOP consisted of both case management and medical services. The MHOP pilot program, which consisted of 20 participants and ran from February 1, 2021 to September 30, 2021, resulted in 86.7% of defendants in permanent housing, with 13.3% in temporary housing while awaiting a permanent home. After the pilot, 73.3% were receiving disability benefits, and 26.7% of patients had benefits pending. The total cost for the 20 pilot participants in 2020 was $362,218, whereas in 2021, before entry into MHOP, it was $57,748. After entry into MHOP, the community costs were $12,631. The monthly average arrest rate dropped 81% for pilot participants [4].

Rational for programmatic redesign

The program continued to expand and run in an ACT-like model until 2025, when the Health Resources and Services Administration (HRSA) underwent significant restructuring as part of a Department of Health and Human Services (HHS) plan to merge with SAMHSA into a new "Administration for a Healthy America" [5]. This period, beginning April 2025, was marked by substantial staffing cuts and funding shifting, requiring changes to Sulzbacher's Behavioral Health Department staffing. While typical ACT ratios rely on one 40-hour psychiatrist for every 100 patients, Sulzbacher had to decrease the staffing ratio to one 10-hour psychiatrist for 80 patients.

To address the needs of the MHOP participants while still allowing for flexibility to access clinical services, the MHOP psychiatrist implemented Open Access Scheduling (OAS) beginning May 2025. OAS schedules are modeled after the scheduling systems used in urgent care clinics, where no appointments are scheduled, and patients are seen on a walk-in basis. With OAS systems, there is a degree of flexibility with the schedule [6]. This type of system has been studied in outpatient primary care offices and has shown some positive benefits related to provider productivity [7]. One community mental health center implemented an OAS system in rural Northeast Tennessee and Southwest Virginia and found a decrease in the no-show and cancellation rates in the clinics and more consistent improvement in the cancellation rates [6]. One Kaiser Permanente psychiatrist implemented OAS in their outpatient clinic and noted greater than 70% utilization rates for both days a walk-in model was offered compared to patients seen under a traditional scheduling system [8]. One hospital-based primary care office conducted a study where patients determined to be at high risk of missing an initial psychiatric evaluation, based on previously missed medical visits, were triaged to a walk-in style access to care with a notable increase in the ratio of filled to initial booked appointments [9]. Given the positive outcomes in the aforementioned studies, MHOP sought to investigate whether OAS had an effect on the rates of missed long-acting injectable (LAI) antipsychotics, overdue psychiatric appointments, and incarceration.

## Materials and methods

The study was a retrospective comparative analysis conducted among participants of the MHOP through the Sulzbacher Community Health Center in Jacksonville, Florida. The proposed duration of the investigation was roughly 24 months, from April 2024 to May 2026, comprising 12 months before and after implementation of OAS. The University of Florida Department of Research Operations granted IRB exemption status on June 26, 2026 (protocol number ET00054707), allowing for retrospective data collection and analysis. A total of 122 participants were enrolled in MHOP at the time the study period began in April 2024. Inclusion criteria were adults 18 years of age or older enrolled in MHOP, diagnosed with a serious mental illness (schizophrenia, bipolar disorder), and had at least one documented clinical encounter. Exclusion criteria included those without a documented diagnosis of a primary thought or mood disorder, not enrolled in MHOP at the time the study period began, and who were less than 18 years of age. 

Deidentified data, obtained from electronic medical records, of the number of both overdue LAI antipsychotics and psychiatric appointments and publicly available arrest rates for MHOP participants, 12 months before and 12 months after implementation of OAS, were uploaded to a Microsoft Excel spreadsheet (Microsoft Corp., Redmond, WA, USA). The data were paired side by side based on the timelines of before and after OAS implementation, which was occurred in May 2025, the first 12 months from May 6, 2024 to April 30, 2025 and the latter 12 months from May 5, 2025, to April 27, 2026. The data were then expressed as percentages based on the total number of visits scheduled for that week, e.g. Week 1 followed by Week 2 and so forth up to Week 52. Excel Data Analysis ToolPak (Microsoft Corp., Redmond, WA, USA) was used to run a paired t-test comparing the mean values before and after the implementation of OAS, with an alpha of 0.05. An unpaired t-test was used to compare the arrest rates 12 months before and after implementation of the OAS model.

## Results

**Figure 1:**
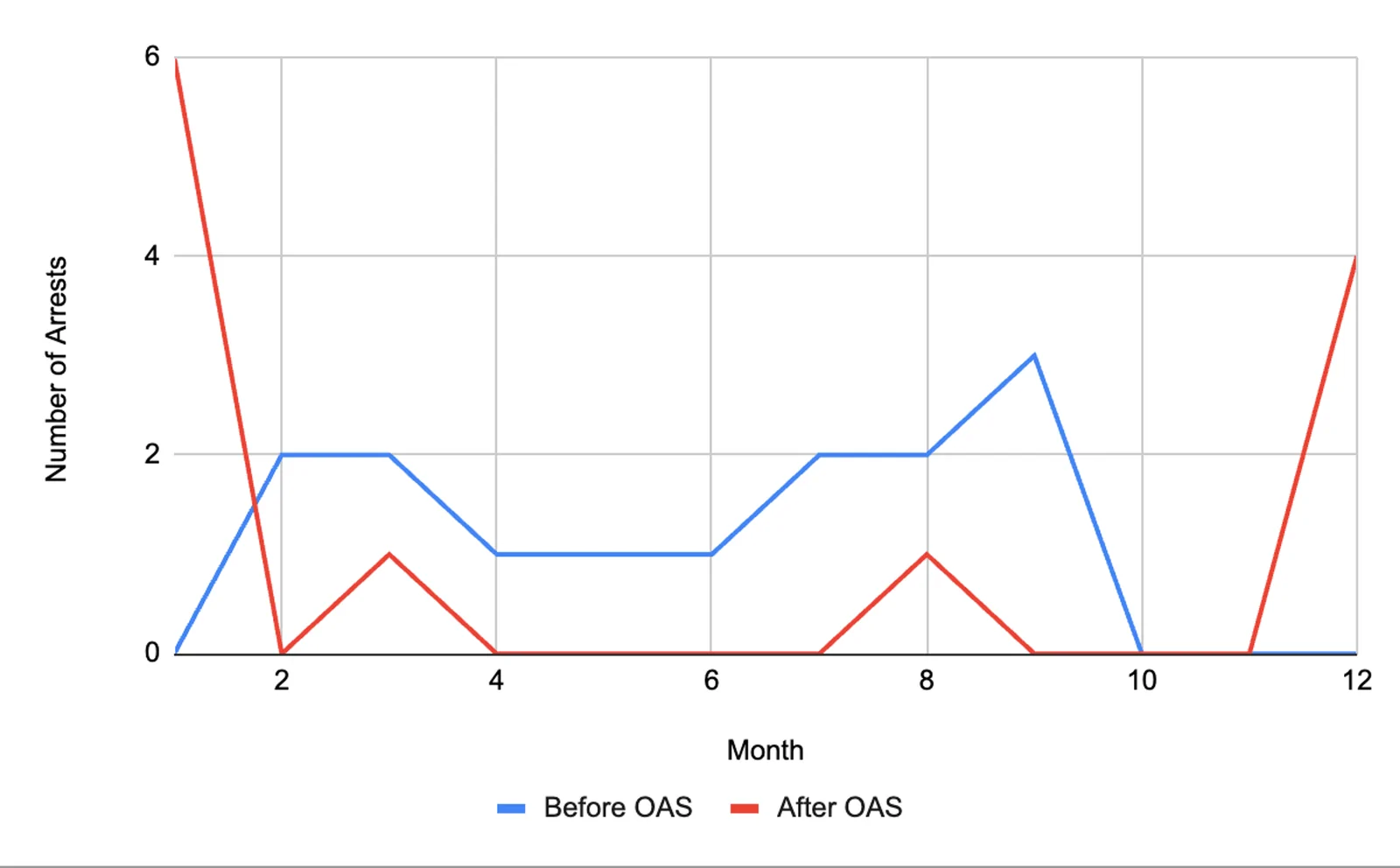
Number of arrests by month. OAS: Open Access Scheduling.

**Figure 2:**
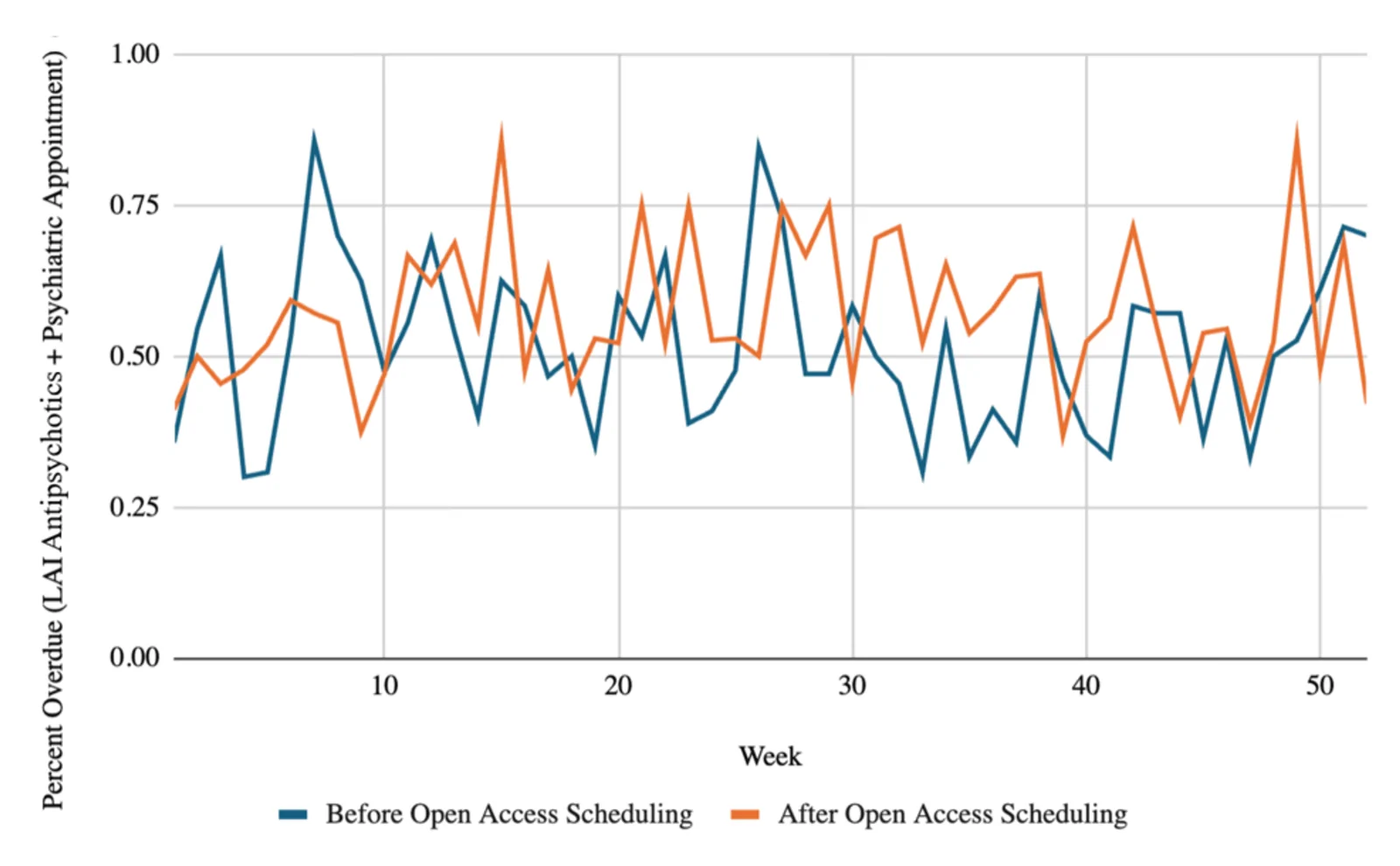
Number of missed LAI antipsychotics and overdue psychiatric appointments. LAI: long-acting injectable.

**Figure 3:**
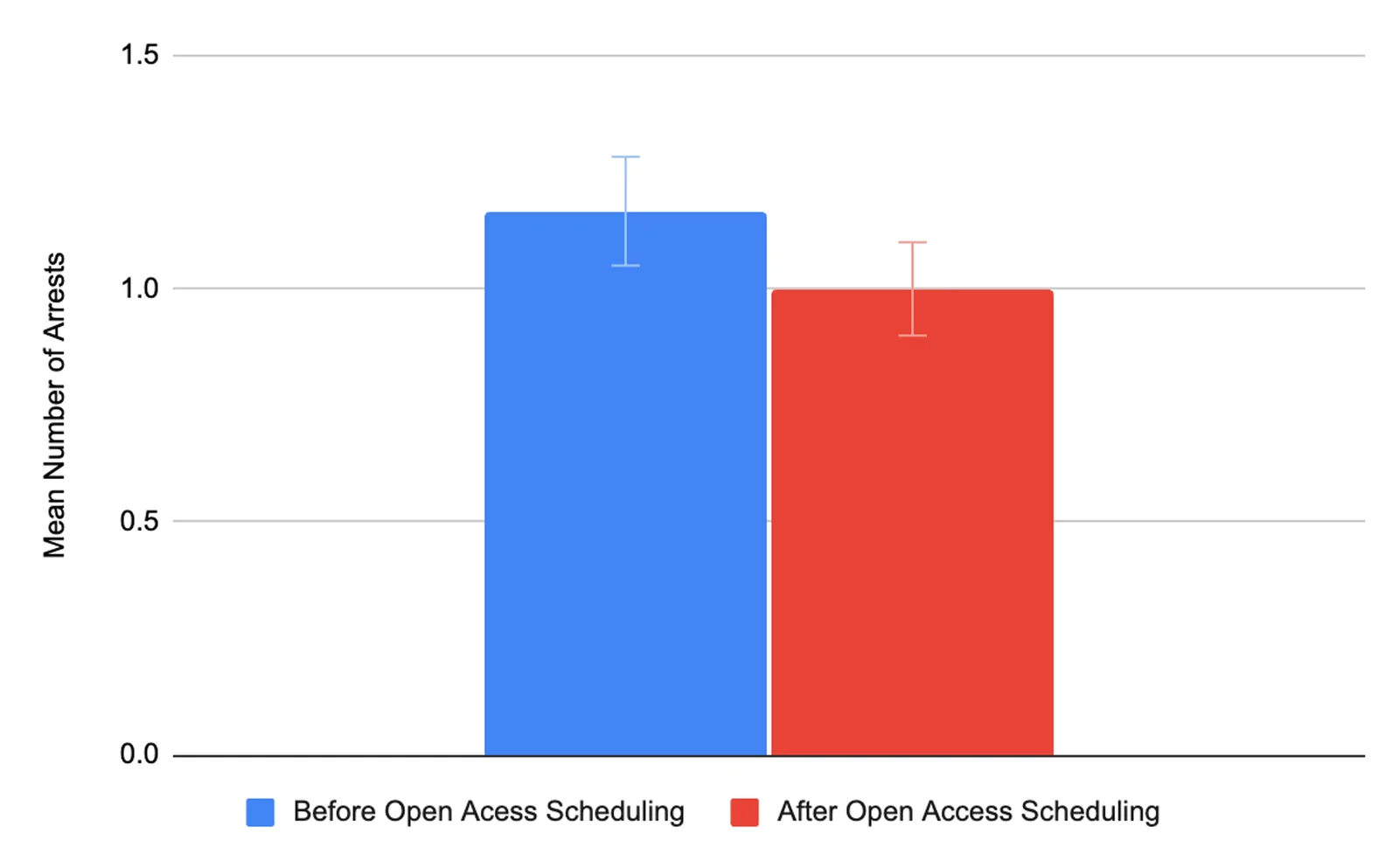
Mean arrest rates before and after the implementation of OAS. OAS: Open Access Scheduling.

The mean difference between the percentage of overdue LAI antipsychotics and missed appointments before and after implementation of OAS yielded (*t*(51) = 2.23, p = 0.032). The mean difference was positive and statistically significant, suggesting higher rates of missed LAI antipsychotics and overdue psychiatric appointments. The mean difference between arrest rates before and after implementation of OAS yielded (*t*(17) = 0.26, p = 0.796) and thus not statistically significant. Figures 1, 2 illustrate the number of arrests and missed LAI antipsychotics and appointments. Figure 3 illustrates the mean number of arrests before and after implementation of OAS.

## Discussion

With recent federal-level budget cuts through the Department of HHS and the subsequent strain on staffing ratios at MHOP, a new model was proposed, OAS. The purpose of this retrospective analysis was to compare if OAS had an impact on the number of missed LAI antipsychotics and overdue psychiatric appointments as well as the rates of incarceration. The results of this study, illustrating higher rates of missed LAI antipsychotics and overdue psychiatric appointments, may have been influenced by confounding factors such as changes in patient acuity and resource availability. A limitation of this study includes decreased generalizability to other settings as individuals were primarily receiving care at a single site. Strengths of this study include the assessment of clinically meaningful outcomes such as the rate of LAI antipsychotics and a retrospective design that enhanced the ability to investigate an uncommon, and often understudied, population.

## Conclusions

Untreated severe mental illness, even in communities with robust health systems, leaves persons at increased vulnerability for risk of hospitalization and incarceration. The ACT model historically provided a tailored approach of meeting individuals with severe mental illness where they are and to lessen the psychosocial difficulties towards receiving care such as those lacking modes of transportation, a support system, and financial means to pay for care. As noted earlier, funding for ACT and resource availability varies across communities, and Sulzbacher sought to investigate an alternative approach to healthcare access through OAS. Given the success of the aforementioned studies on OAS, Sulzbacher hypothesized that this model may improve certain health outcomes relevant to those suffering from serious mental illness. A retrospective data analysis showed a statistically significant worsening of missed LAI antipsychotics and overdue psychiatric appointments after implementation of OAS; however, the mean number of arrests before and after OAS was roughly the same. Recommendations for future studies include larger sample sizes and longer duration of investigation. Nonetheless, this study emphasizes the continued need for consistent and adequate funding to serve those who are historically marginalized members of the community.
